# Supplementary material for: Comprehensive Annotation and Functional Exploration of MicroRNAs in Lettuce
Source: Front Plant Sci. 2021 Dec 24;12:781836. doi: 10.3389/fpls.2021.781836 (PMC8739914; doi:10.3389/fpls.2021.781836)
Supplement: Supplementary file 2 [file Data_Sheet_8.ZIP › GSM2230750.plot/Lsa-miR171a_Lsat_1_v5_gn_1_27961.1_2558_TPlot.pdf]

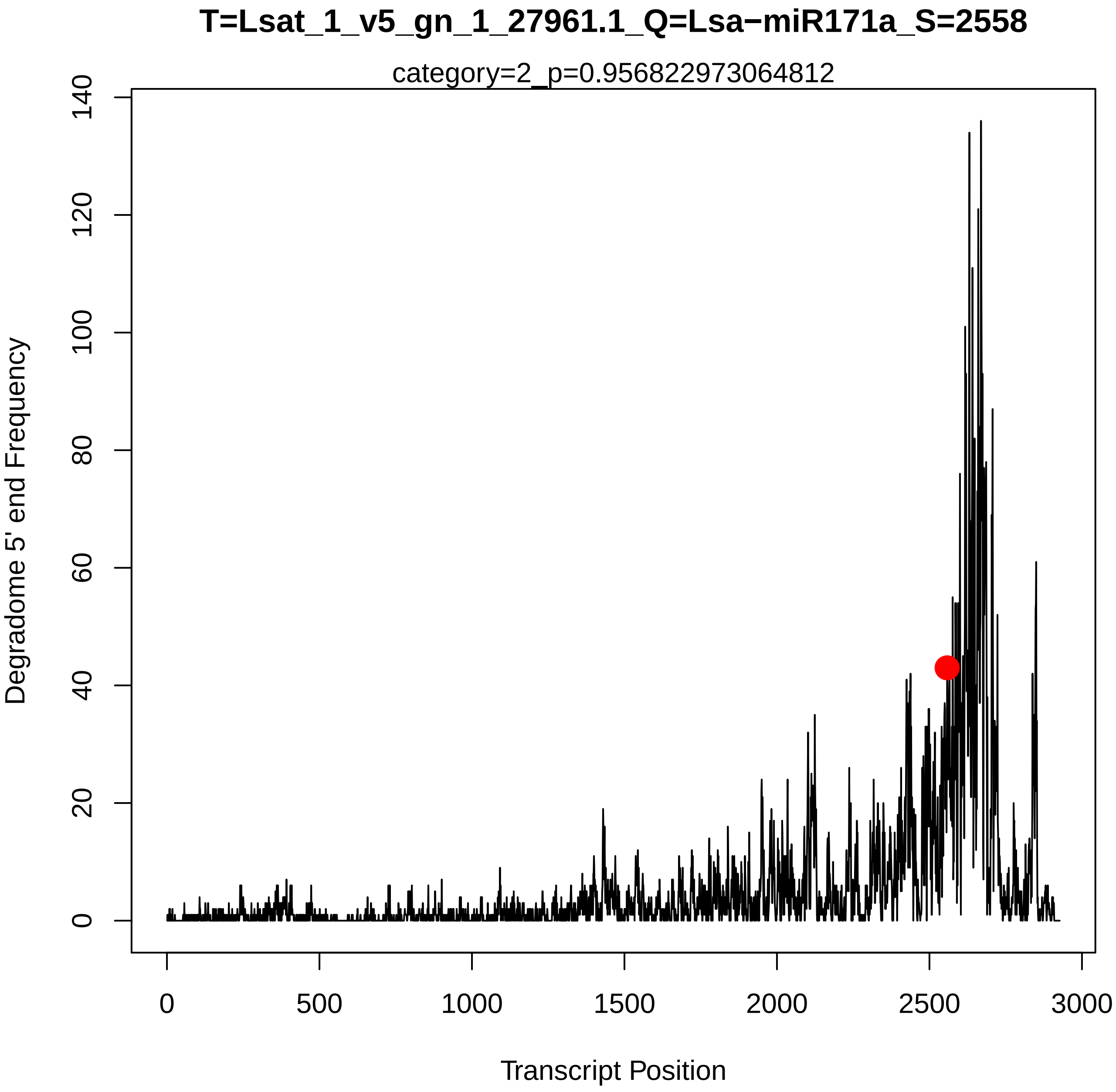

T=Lsat_1_v5_gn_1_27961.1_Q=Lsa−miR171a_S=2558
category=2_p=0.956822973064812
Degradome 5' end Frequency
Transcript Position
0
20
40
60
80
100
120
140
0
500
1000
1500
2000
2500
3000
